# ApoB100 and Atherosclerosis: What’s New in the 21st Century?

**DOI:** 10.3390/metabo14020123

**Published:** 2024-02-12

**Authors:** Dimitris Kounatidis, Natalia G. Vallianou, Aikaterini Poulaki, Angelos Evangelopoulos, Fotis Panagopoulos, Theodora Stratigou, Eleni Geladari, Irene Karampela, Maria Dalamaga

**Affiliations:** 1 Second Department of Internal Medicine, Hippokration General Hospital, School of Medicine, National and Kapodistrian University of Athens, 11527 Athens, Greece; dimitriskounatidis82@outlook.com; 2 Department of Internal Medicine, Evangelismos General Hospital, 10676 Athens, Greece; fotis_1992@hotmail.com (F.P.); elgeladari@gmail.com (E.G.); 3 Hematology Unit, Second Department of Internal Medicine, School of Medicine, National and Kapodistrian University of Athens, 11527 Athens, Greece; aikaterini.poulaki@gmail.com; 4 Roche Diagnostics Hellas S.A., 15125 Athens, Greece; angelos.evangelopoulos@roche.com; 5 Department of Endocrinology and Metabolism, Evangelismos General Hospital, 10676 Athens, Greece; theodorastratigou@yahoo.gr; 6 Second Department of Critical Care, Attikon General University Hospital, Medical School, National and Kapodistrian University of Athens, 12462 Athens, Greece; eikaras1@gmail.com; 7 Department of Biological Chemistry, Medical School, National and Kapodistrian University of Athens, 11527 Athens, Greece; madalamaga@med.uoa.gr

**Keywords:** ApoB100, ApoB48, ApoB/ApoA1 ratio, atherosclerosis, cardiovascular risk, chronic low-grade inflammation, metabolic syndrome, triglyceride-rich lipoproteins

## Abstract

ApoB is the main protein of triglyceride-rich lipoproteins and is further divided into ApoB48 in the intestine and ApoB100 in the liver. Very low-density lipoprotein (VLDL) is produced by the liver, contains ApoB100, and is metabolized into its remnants, intermediate-density lipoprotein (IDL) and low-density lipoprotein (LDL). ApoB100 has been suggested to play a crucial role in the formation of the atherogenic plaque. Apart from being a biomarker of atherosclerosis, ApoB100 seems to be implicated in the inflammatory process of atherosclerosis per se. In this review, we will focus on the structure, the metabolism, and the function of ApoB100, as well as its role as a predictor biomarker of cardiovascular risk. Moreover, we will elaborate upon the molecular mechanisms regarding the pathophysiology of atherosclerosis, and we will discuss the disorders associated with the APOB gene mutations, and the potential role of various drugs as therapeutic targets.

## 1. Introduction

Apolipoprotein B (ApoB) is a crucial structural protein component found in all major atherogenic lipoproteins. The ApoB lipoprotein occurs in two isoforms, namely, ApoB48 and ApoB100. ApoB48 is synthesized in the intestine and is present in chylomicrons and their remnants. On the other hand, ApoB100 is secreted by the liver and serves as a key component in LDL, IDL, VLDL, and Lp(a) [1]. Although both forms of the ApoB lipoprotein are encoded by the APOB gene, they exhibit distinct effects in lipid metabolism. The ApoB100 gene comprises 28 introns and 29 exons and is located on chromosome 2. Mutations in the APOB100 gene may contribute to the development of premature atherosclerosis [2].

Atherosclerosis (AS) is a chronic and progressive inflammatory process considered a major cause of global mortality and morbidity, accounting for approximately 50% of deaths in Western countries [3]. The deposition and retention of ApoB-containing particles in the subendothelium are crucial in the initiation of the pro-atherosclerotic process. Additionally, chronic inflammation and immune dysregulation appear to accelerate atherosclerosis development, predisposing individuals to the formation of vulnerable atherosclerotic plaques susceptible to rupture and erosion. Consequently, recent research has focused on the development of novel agents that may potentially attenuate the activation of the inflammatory cascade, leading to a more favorable atherosclerotic milieu [4].

Traditionally, the measurement of LDL-C is considered the gold standard method for evaluating atherosclerotic cardiovascular disease (ASCVD) risk and assessing the effectiveness of lipid-modifying therapies. In contrast, ApoB levels reflect the total amount of atherogenic lipoproteins, as each VLDL, LDL, IDL, and Lp(a) particle exclusively contains one ApoB molecule. Thus, ApoB may serve as a superior predictor of ASCVD burden, particularly in individuals with residual CVD risk who do not achieve therapeutic goals despite receiving appropriate lipid-lowering treatment. Nowadays, international guidelines have incorporated ApoB measurement as an alternative to the LDL-C assay, especially in subjects with very low LDL-C levels or in patients with metabolic syndrome (METs) [5].

Although this manuscript is not a systematic review, for the preparation of this narrative review, we applied the MESH search terms “ApoB100 and cardiovascular disease” in the PubMed NIH database from inception for the past 10 years until 1st February 2024, which yielded 209 outputs. Among the 209 manuscripts, 39 were excluded in total. More specifically, eight manuscripts dealt with chemistry issues regarding the measurement of ApoB100, eight were associated with probiotics, prebiotics, and the gut microbiota, five manuscripts were associated with brain and/or neurogenerative disorders, four articles were associated with rheumatic/bone diseases, four concerned pediatric patients, three manuscripts were related to hypertension, and one was a book and not an article. Furthermore, four manuscripts were written in Russian and one in Czech. Therefore, among the 209 manuscripts, a total of 170 manuscripts were included in this literature search. We also acknowledge that not all of these manuscripts can be covered in the context of this review.

## 2. ApoB100: Structure, Function, and Metabolism

The ApoB100 glycoprotein is composed of 4536 amino acid residues, with a molecular weight exceeding 500,000 Daltons. It possesses several hydrophobic domains that function as lipid-binding areas, along with binding sites for the LDL receptor (LDL-R) and heparin-like molecules. A secondary structure analysis has revealed that the ApoB100 particle adopts a pentapartite structure, consisting of five domains: βα1, β1, α2, β2, and α3. In this context, the term “α” denotes a predominantly α-helical structure, while “β” signifies a preponderantly β-sheet domain. The β-sheet regions play a crucial role in establishing irreversible strong bonds to the lipid core [6,7].

The assembly of ApoB100 occurs in the liver and is facilitated by the first 884 amino acid residues of the lipoprotein, involving the microsomal triglyceride transfer protein (MTP). MTP functions as an endoplasmic reticulum (ER) resident chaperone, aiding in the formation of ApoB within ribosomes. Specifically, MTP facilitates the transfer of triglycerides and cholesterol esters to nascent ApoB particles, which then translocate to the lumen of the ER [8,9]. Notably, a unique aspect of this process is that 50–80% of the newly synthesized ApoB100 polypeptide undergoes degradation in the liver before secretion. The primary determinants of this modification include the availability of major lipoprotein lipids (triglycerides, cholesteryl esters, and phospholipids) and the functional adequacy of MTP [10].

ApoB100 plays a pivotal role in the assembly and production of very low-density lipoprotein (VLDL) by the liver. VLDL is composed of approximately 90% lipids and 10% proteins. Among its total lipid content, triglycerides constitute 70% of the lipoprotein’s mass, while cholesterol esters and fatty acids make up the remaining 30%. Each VLDL particle contains one ApoB100 molecule, thereby influencing the production of VLDL and triglycerides by hepatocytes. As previously mentioned, the presence of MTP is vital for ApoB100 lipidation and the formation of nascent VLDL. Nascent VLDL is then secreted into circulation and interacts with high-density lipoprotein (HDL) [11].

VLDL acquires ApoCII and ApoE lipoproteins from circulating HDL, undergoing transformation into mature VLDL. Specifically, ApoCII and ApoCIII are transferred from HDL to VLDL, activating or inhibiting lipoprotein lipase (LPL). Additionally, VLDL receives ApoE from HDL to facilitate the clearance of triglyceride-enriched remnants and intermediate-density lipoprotein (IDL). Cholesteryl ester transfer protein (CETP) supports the transfer of cholesteryl esters from HDL to VLDL in exchange for triglycerides to HDL [12]. Moreover, HDL directly transfers cholesterol to the liver through its interaction with the scavenger receptor class B type I (SR-BI). The hepatic SR-BI protein is essential in cholesterol homeostasis and lipoprotein metabolism, enhancing reverse cholesterol transport (RCT) through the selective uptake of HDL. Ultimately, mature VLDL transports endogenous lipids, predominantly triglycerides, from the liver to peripheral tissues [13].

Initially, plasma VLDL is metabolized to cholesteryl ester-enriched intermediate-density lipoprotein (IDL) and low-density lipoprotein (LDL) molecules. This is achieved through the hydrolysis of triglycerides by lipases, involving lipoprotein lipase (LPL) and hepatic lipase (HL). LDL is the main cholesterol-transferring lipoprotein in plasma, typically accounting for at least 70% of the total serum cholesterol. ApoB100 is the only protein contained in the LDL particle and works as a ligand for the LDL receptor (LDL-R) via specific areas in the ApoB100 molecule. LDL-binding domains are located in the amino acids 3100–3400, allowing the specific uptake of LDL by the LDL-Rs [14,15]. Once the LDL-R recognizes ApoB100 on LDL, a receptor–lipoprotein complex is formed, followed by cellular internalization via endocytosis. Subsequently, LDL is recycled, whereas ApoB100 lipoprotein endures lysosomal degradation to amino acids and unesterified cholesterol [10]. Apart from the LDL-R binding domains, ApoB100 exhibits at least eight possible proteoglycan (PG)-binding sites. The interplay between ApoB100 and proteoglycans seems to be the primary mechanism responsible for the retention of the LDL particles in the subendothelium, which is the initial step of the atherosclerotic process [16].

**Figure 1:**
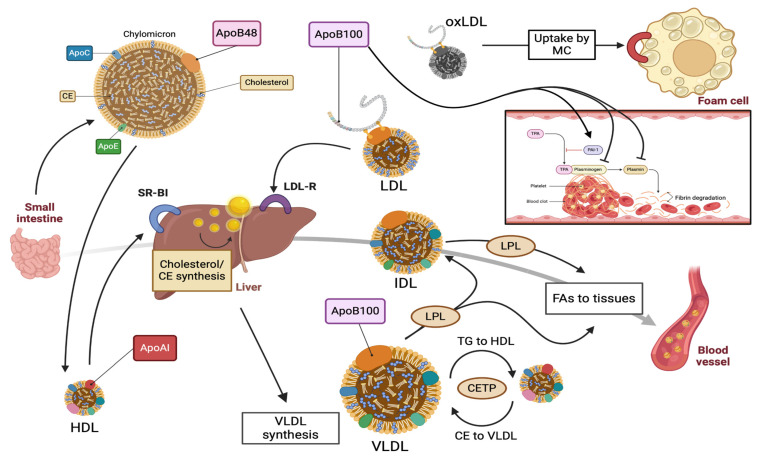
Schematic presentation of ApoB100-containing lipoprotein metabolism. Dietary fat is absorbed and incorporated into triglyceride-rich chylomicrons, which are released into circulation. ApoB48 is a distinctive protein found exclusively in chylomicrons. Subsequently, LPL hydrolyzes the triglycerides within chylomicrons, leading to the formation of remnants. These remnants are then secreted as VLDL particles by the liver. Meanwhile, CETP facilitates the transfer of cholesteryl esters from HDL to VLDL in exchange for triglycerides. HDL aids in cholesterol transfer to the liver via interaction with SR-BI. Plasma VLDL undergoes further metabolism, transforming into IDL and LDL particles through triglyceride hydrolysis mediated by LPL and HL, respectively. IDL can either be taken up by hepatocytes or, under the influence of HL, converted into LDL. ApoB100 acts as a ligand for the hepatic LDL-R-mediated clearance of LDL from the bloodstream. LDL, when oxidized, provokes foam cell formation. Additionally, ApoB100 is a crucial component of Lp(a), which bears structural similarities to plasminogen and is potentially implicated in thromboembolic risk [15,16,17]. Abbreviations: ApoA1: apolipoprotein A1; ApoB48: apolipoprotein B48; ApoB100: apolipoprotein B100; ApoC: apolipoprotein C; ApoE: apolipoprotein E; CE: cholesteryl esters; CETP: cholesteryl ester transfer protein; FAs: fatty acids; HDL: high-density lipoprotein; HL: hepatic lipase; IDL: intermediate-density lipoprotein; LDL: low-density lipoprotein; LDL-R: low-density lipoprotein receptor; LPL: lipoprotein lipase; MC: macrophage; ox-LDL: oxidized LDL; PAI-1: Plasminogen Activator Inhibitor-1; SR-BI: Scavenger Receptor class B, type I; TG: triglycerides; TPA: Tissue Plasminogen Activator; VLDL: very low-density lipoprotein. Created with BioRender.com.

Apolipoprotein B100 plays a central role in lipoprotein(a), where an ApoB100-LDL particle covalently links to the heavily glycosylated and polymorphic glycoprotein apo(a). Lp(a) is widely believed to possess both pro-atherogenic and thrombotic properties, acting as the main transporter of oxidized phospholipids (Ox-PL) in plasma. Lp(a) primarily acquires oxidized phospholipids from LDL, contributing to the deposition of lipids in the arterial intima. Traditionally, Lp(a) is thought to exhibit prothrombotic characteristics due to its structural similarity to the fibrinolytic pro-enzyme plasminogen. This similarity interferes with the function of plasminogen activator inhibitor-1 (PAI-1), a member of the serpin superfamily and the primary inhibitor of plasminogen activation. Specifically, PAI-1 inactivates urokinase-type plasminogen activator (UPA) and tissue-type plasminogen activator (TPA), preventing the conversion of plasminogen to plasmin [17]. However, the involvement of Lp(a) in the atherothrombotic burden has become a subject of debate in recent years. While a significant number of early reports advocate a positive correlation between Lp(a) levels and the risk of venous thromboembolism, recent genetic evidence suggests that this association is present only at very high Lp(a) concentrations. Even if Lp(a) is not intrinsically antifibrinolytic, its impact on thrombosis formation, through effects on platelets and coagulation, remains to be elucidated [18,19]. Figure 1 provides a schematic presentation of the metabolism of ApoB100-containing lipoproteins.

## 3. Molecular Mechanisms Regarding the Pathophysiology of Atherosclerosis

Atherosclerosis is a progressive, chronic inflammatory process characterized by arterial stiffness resulting from the accumulation and retention of ApoB-containing LDL particles within the arterial wall. Over time, the intima and media layers of the arteries exhibit extracellular matrix protein deposition and calcification. The hallmark of the atherosclerotic process is plaque formation, which has the potential to lead to lumen stenosis and thrombosis. The formation of the atheromatous plaque is a chronic process marked by the accumulation of oxidized LDLs (ox-LDLs), the recruitment of inflammatory cells, and the deposition of adhesion molecules, chemoattractants, and growth factors.

Specifically, retained LDL molecules undergo modification through oxidation, giving rise to the formation of oxidized LDL (ox-LDL). Ox-LDL serves as the initial stimulus for the inflammatory cascade, recognized by danger-associated molecular patterns (DAMPs). Toll-like receptors (TLRs) play a pivotal role, as oxidatively modified LDL particles enhance the endothelial cell expression of leukocyte adhesion and recruitment molecules such as E-selectin, vascular cell adhesion molecule (VCAM-1), and intercellular adhesion molecule-1 (ICAM-1). These molecules bind to their ligands expressed on white blood cells [20,21].

Monocytes constitute the predominant leukocyte cell type within the plaque. Upon infiltration of the arterial wall, they undergo conversion into macrophages facilitated by monocyte-colony stimulating factor (M-CSF). Activated macrophages contribute to the inflammatory milieu by undergoing a phenotypic switch from the M2 to M1 phenotype. The M1 macrophages release pro-inflammatory cytokines (e.g., TNF-α and IL-1β), further amplifying LDL-driven inflammation. Oxidized LDL (Ox-LDL) molecules interact with scavenger receptors in macrophages (e.g., CD36 and LOX-1), leading to their differentiation into foam cells. As lipid-laden macrophages or foam cells are formed, the process continues with central necrosis, intraplaque hemorrhages, fibrosis due to the activation of smooth muscle cells, and the deposition of extracellular matrix, as well as calcification. In advanced stages, when the atherogenic plaque becomes highly inflammatory with increased central necrosis, hemorrhage, and particularly a thin fibrous cap, it becomes vulnerable to rupture. The combination of these characteristics, along with hemodynamic stress, renders the atheromatous plaque susceptible to rupture [22]. Furthermore, dendritic cells internalize ox-LDLs, subsequently promoting antigen presentation to T-cells by both macrophages and dendritic cells. Consequently, the adaptive immune system is activated within the atherosclerotic lesion [23]. 

Immune modulation plays a fundamental role in the development of atherosclerosis. Innate immune activation induces the production of leukocyte adhesion molecules and chemotactic factors, leading to the enrichment of atherosclerotic lesions with effector CD4+ T cells. CD4+ T cells can be further categorized into conventional T helper (Th) cells and regulatory T (Treg) cells. Th cells control adaptive immunity against pathogens, while Treg cells counterbalance the potentially deleterious effects of Th cells. Specifically, Th1 cells promote atherosclerosis by producing pro-inflammatory cytokines such as IL-2, TNF-α, and IFN-γ, sustaining chronic inflammation and contributing to foam cell formation. Additionally, Th17 cells aid in fibrosis and potentially plaque stabilization through the production of IL-17. In contrast, effector T cells of the Treg subtype exert atheroprotective properties by mitigating immune response and inflammation. Ultimately, the defective phagocytosis of lipid-laden apoptotic macrophages in the atherosclerotic lesion contributes to enhanced inflammation [24].

CD4+ Th cells within atheromatous plaques serve as antigen-presenting cells (APCs) and contribute to atherosclerosis by producing pro-inflammatory cytokines through an MHC-restricted process. Furthermore, CD4+ T cells in human plaques recognize oxidized LDL by binding to MHC-presented peptide epitopes derived from ApoB-100 [25]. This observation suggests that T cells within the atheroma plaque may eventually identify peptide motifs from native LDL molecules and its ApoB100 component [26]. The immune response to these ApoB epitopes is proposed to be associated with the severity of coronary artery disease [27]. Interestingly, ApoB+ T cells are suggested to form an oligoclonal population in the lymph nodes of healthy mice, displaying a Treg-like transcriptome. These cells undergo an increase in atherosclerosis and gradually transform into pathogenic mixed TH1/TH17 phenotype cells with pro-inflammatory characteristics [28].

Changes in the atherosclerotic environment can also be influenced by the presence of specific bioactive lipids. These lipid mediators may arise from intracellular signaling pathways, utilizing cellular membrane phospholipids as substrates. Alternatively, they may result from the extracellular metabolism of phospholipids, with circulating lipoproteins being the primary source of their origin. Specifically, intracellular cytosolic phospholipase A_2_ (PLA_2_) metabolizes phospholipids into fatty acids, leading to the release of arachidonic acid and lysophospholipids. Arachidonic acid is involved in the cyclooxygenase and lipoxygenase/leukotriene pathways, facilitating the interaction of leukotrienes, prostaglandins, lipoxins, and thromboxane with specific extracellular receptors. Importantly, PLA_2_ isoenzymes are suggested to enhance LDL atherogenicity by modifying LDL, involving the formation of lysophosphatidylcholine [29].

Intracellular signaling pathways, such as the p38 Mitogen-Activated Protein Kinase and Janus kinase/STAT pathway, are implicated in the inflammatory process by amplifying the production of pro-inflammatory cytokines [30]. Oxidative stress and the activation of the nuclear factor-kappa B (NF-κB) are also involved. Specifically, reactive oxygen species (ROS) have been demonstrated to induce endothelial dysfunction due to the dysregulation of nitric oxide (NO). Additionally, the activation of NF-κB modulates the expression of adhesion molecules, such as IL-8 and VCAM-1, contributing to the recruitment of circulating mononuclear leukocytes to the arterial intima. Activated NF-κB has been identified at various locations within the vulnerable plaque and at different stages of the atherosclerotic process [31,32]. Remarkably, ROS may enhance the activation of NF-κB through the phosphorylation of its transcriptional p65 subunit. Along with the p50 subunit, p65 triggers the formation of inflammatory cytokines while simultaneously promoting monocyte adhesion, endothelial dysfunction, apoptosis, and oxidative stress [33].

**Figure 2:**
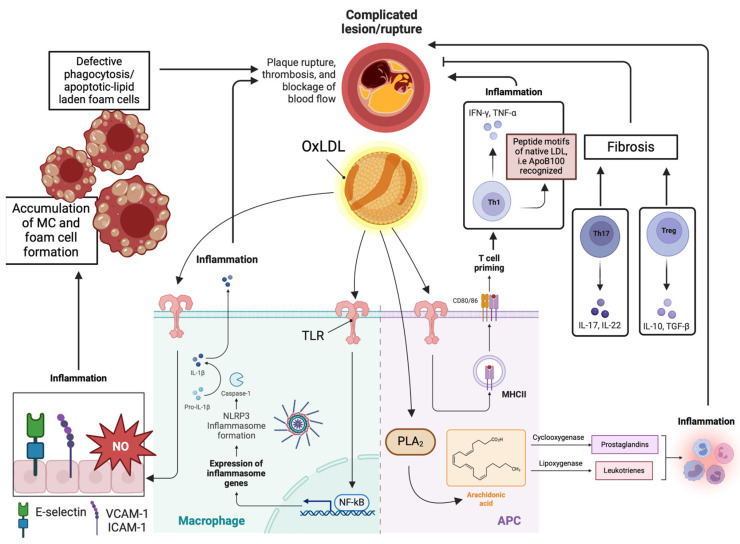
Main molecular mechanisms involved in atherosclerosis. Abbreviations: APC: antigen-presenting cell; ICAM-1: Intercellular Adhesion Molecule-1; IFN-γ: interferon-γ; IL-1β: interleukin-1β; IL-10: interleukin-10; IL-17: interleukin-17; IL-22: interleukin-22; MHCII: major histocompatibility complex class II; NF-κB: nuclear factor kappa-light-chain-enhancer of activated B cells; NO: nitric oxide; Ox-LDL: oxidized-LDL; PLA2: phospholipase A2; TGF-β: transforming growth factor beta; Th1: T helper 1 cells; Th17: T helper 17 cells; TLR: Toll-like receptor; TNF-α: tumor necrosis factor-α; VCAM-1: vascular cell adhesion molecule-1. Created with BioRender.com.

In recent years, the key role of the NLRP3 inflammasome in the pathogenesis of atherosclerosis has gained prominence. The oxidation of LDL particles leads to the generation of cholesterol crystals within the plaque. Cholesterol accumulation and crystallization in macrophages, coupled with the release of danger-associated molecular patterns (DAMPs) from stressed and dying cells, may stimulate the NLRP3 inflammasome, eventually resulting in the production of IL-1β and/or IL-1α [34]. The clarification of the underlying mechanisms responsible for the activation of the inflammatory cascade in atherosclerosis is of the utmost importance as it may provide insights into the development of novel and beneficial therapeutic approaches. The key molecular mechanisms that intervene in the atherosclerotic process are illustrated in Figure 2.

## 4. Novel Predictors of Cardiovascular Burden: The Role of ApoB100

While the measurement of LDL-C has traditionally served as the gold standard for assessing ASCVD risk, there are limitations to relying solely on this metric. A considerable percentage of individuals, even those undergoing efficient lipid-lowering treatments, may experience ASCVD-related events despite having normal or low LDL-C levels. This phenomenon is attributed to residual cardiovascular disease risk, particularly in the presence of metabolic syndrome, where underlying inflammation and metabolic impairment play crucial roles. In response to these limitations, recent years have seen the establishment of novel indices that encompass all ApoB-containing lipoproteins. Two key parameters in this context are ApoB and non-high-density lipoprotein cholesterol (non-HDL-C). These indices may provide a more comprehensive assessment of the atherogenic lipoproteins and offer additional insights beyond LDL-C measurements.

### 4.1. ApoB100

The deposition and retention of ApoB-containing lipoproteins in the subendothelium are essential for initiating the pro-atherosclerotic process. The concentration of ApoB is indicative of the total amount of atherogenic lipoproteins, as each VLDL, LDL, IDL, and Lp(a) particle contains precisely one ApoB molecule. Importantly, this association remains independent of particle density and the heterogeneity of its cholesterol composition. Additionally, the lipid content of the particles appears to reflect the proportion of circulating atherogenic lipoproteins restrained within the arterial lumen [35]. Therefore, ApoB may be considered a superior index, compared to LDL-C, for evaluating atherosclerotic cardiovascular risk, particularly in individuals with residual cardiovascular disease risk despite appropriate treatment.

In most cases, ApoB levels reflect the number of LDL particles, with over 90% of circulating ApoB-containing lipoproteins being LDL particles. However, this pattern differs in individuals with coronary heart disease or metabolic syndrome, as highly atherogenic small and dense LDL particles (sd-LDL) are observed [36,37]. Patients with myocardial infarction, diabetes mellitus, or hypertriglyceridemia may experience major cardiovascular events despite achieving the recommended LDL-C goals. Compared to larger LDL particles, sd-LDL subfractions contain less cholesterol. Additionally, they can penetrate the arterial wall, acting as a source of cholesterol and lipid storage, and they tend to linger longer in circulation due to their low affinity towards the LDL-R. Consequently, they are highly susceptible to chemical modification, glycosylation, and oxidation [38].

ApoB, non-HDL-C, and LDL-C are independent predisposing factors for CVD risk. However, accumulating evidence highlights the superiority of ApoB over LDL-C in predicting CVD risk. Therefore, even in cases presenting with normal LDL-C, ApoB measurement can provide valuable information for assessing ASCVD. The Apoprotein-related Mortality Risk Study (AMORIS) is arguably the most important study regarding the superiority of ApoB100 over LDL-C in predicting the risk of fatal myocardial infarction. The results of this study underscored the precedence of ApoB at every level of cholesterol, with the difference with LDL-C being most notable in the lower half of the distribution [39]. Similar results have also been reported in subjects with type 2 diabetes mellitus. Lim et al. demonstrated that in T2DM patients, ApoB may be significantly and independently associated with metabolic syndrome, making it an effective predictor of CVD risk in these cases [40]. 

Recently, Marston et al. presented the results of a prospective cohort study comparing 389,529 participants in the primary prevention group with 40,430 individuals with atherosclerosis on statin therapy. In the primary prevention cohort, triglycerides, ApoB, and non-HDL-C were each individually related to incident myocardial infarction (MI). However, when these three lipid indexes were assessed together, only ApoB was correlated with MI in both groups. The authors concluded that the number of ApoB-containing lipoproteins may be superior in reflecting MI risk, independent of the type of lipoprotein or lipid content [41]. Yun et al. reported that ApoB showed the highest adjusted hazard ratio (aHR) per 1-SD for ASCVD, followed by non-HDL-C and LDL-C, after adjusting for age, gender, family history of premature ASCVD, smoking history, hypertension, type 2 diabetes, and chronic kidney disease (CKD) [42]. 

The European Atherosclerosis Society (EAS) and the American Heart Association (AHA) both agree that ApoB measurement may be considered reasonable for cardiovascular risk assessment, particularly in patients with metabolic syndrome, including individuals with obesity, diabetes mellitus, high triglycerides, and very low LDL-C levels [43,44]. In alignment with the Canadian Cardiovascular Society (CCS), LDL-C is recommended as the primary laboratory assay for determining the initiation of statin therapy and as a treatment target for low-, intermediate-, and high-risk patients. However, in cases where serum triglycerides exceed 130 mg/dL, non-HDL-C or ApoB may be used as alternative lipid parameters to LDL-C, according to CCS guidelines [45]. As per the Japanese Atherosclerosis Society (JAS), elevated ApoB levels pose a risk for atherosclerotic cardiovascular disease [46]. 

### 4.2. The ApoB100/ApoA1 Ratio

Unlike ApoB100, ApoA1 constitutes the primary protein component in HDL particles, contributing to approximately 70% of the total apolipoproteins in HDL. ApoA1 is recognized as an anti-atherogenic lipoprotein, primarily influencing reverse cholesterol transport by promoting cholesterol efflux. Additionally, ApoA1 serves as a co-factor for lecithin-cholesterol acyltransferase (LCAT). In turn, LCAT enhances HDL’s cholesterol-carrying capacity by esterifying circulating free cholesterol into cholesteryl esters [47]. Consequently, the ApoB/ApoA1 ratio may reflect the delicate balance between atherogenic and anti-atherogenic lipoproteins in serum, potentially offering a more accurate predictor of cardiovascular risk. Numerous studies have highlighted the role of the ApoB/ApoA1 ratio in assessing cardiovascular risk. Accumulating evidence suggests that lipoprotein ratios may outperform individual lipid parameters as predictors of coronary heart disease, with the ApoB100/ApoA1 ratio identified as an optimal predictor of CHD [48]. In comparison to other traditional risk factors such as smoking, arterial hypertension, and diabetes mellitus, ApoB and the ApoB/ApoA1 ratio have demonstrated strong and independent associations with the risk of acute myocardial infarction [49,50]. Various studies propose a cutoff point of 0.8 for the ratio, signifying an elevated hazard of cardiovascular disease [50,51]. However, Bodde et al. have highlighted that this association appears to be absent in recurrent cardiovascular events during long-term follow-up [52].

In the AMORIS study, a large multicenter investigation exploring the risk of stroke in relation to alterations in pro-atherogenic ApoB lipoprotein particles and anti-atherogenic ApoA1 molecules, elevated ApoB100 and reduced apoA1 levels were associated with a higher risk of stroke, and the Apo ratio emerged as a stronger predictor compared to the LDL-C/HDL-C and total cholesterol/HDL-C ratios [53]. Moreover, the ApoB/ApoA1 ratio emerges as a valuable parameter in predicting the severity of coronary stenosis. In a cross-sectional study involving 792 patients with CHD who underwent coronary angiography, it was found that among various lipid parameters and their ratios, the ApoB100/ApoA1 ratio was the only one positively correlated with the number of stenotic vessels (odds ratio = 3.14, 95% CI = 1.01–6.47, P = 0.048), independently. Additionally, it was identified as a direct mediator between predisposing factors such as age, LDL-C, and obesity, and the burden of CHD. Notably, smoking history and diabetes mellitus were shown to exert a significant impact on the severity of CHD independently of the ApoB100/ApoA1 ratio [54].

Nayak et al. propose that the ApoB100/ApoA1 ratio could serve as an additional parameter, alongside traditional lipid ratios, in assessing cardiovascular risk, particularly in hypertensive patients. Their findings demonstrated a significant increase in the ApoB100/ApoA1 ratio in both stages of hypertension (stage I and stage II) compared to the control group. ApoB100 concentration exhibited a positive correlation with systolic and diastolic blood pressure, with the ApoB100/ApoA1 ratio showing a stronger positive association than the LDL/HDL ratio (r = 0.749, *p* < 0.001, r = 0.756, *p* < 0.001 vs. r = 0.336, *p* < 0.000, r = 0.312, *p* < 0.001) [55]. Recently, Zhan et al. presented the results of a retrospective cohort study involving 860 patients on peritoneal dialysis over a 27-month follow-up period. The authors suggested that an elevated ApoB/ApoA1 ratio may be independently related to cardiovascular disease and all-cause mortality. The highest ApoB/ApoA1 ratio tertile was significantly correlated with a hazard ratio for cardiovascular events of 2.04 (95% CI: 1.21 to 3.44, *p* = 0.008) and for all-cause mortality of 1.60 (95% CI: 1.02 to 2.49, *p* = 0.040) [56].

### 4.3. Non-HDL-C

Non-HDL-C serves as a marker for the cholesterol content of atherogenic ApoB-containing lipoproteins, although it does not account for the number of these particles. Serum non-HDL-C can be calculated using the following formula: non-HDL-C = Total cholesterol − HDL-C. Existing evidence suggests that non-HDL-C is equivalent to LDL-C in ASCVD risk assessment. Moreover, non-HDL-C surpasses LDL-C as it includes remnant cholesterol and remains independent of triglyceride variability. Consequently, in non-fasting samples, as well as in individuals with very low LDL-C levels or very high triglyceride levels, non-HDL-C is considered a more reliable marker compared to LDL-C [57]. Despite this superiority, non-HDL-C is not widely utilized in the evaluation of cardiovascular risk or in assessing the effectiveness of lipid-modifying treatments.

On the contrary, non-HDL-C appears to be less effective than ApoB in predicting ASCVD risk. A meta-analysis of epidemiological studies comparing the relative risks of non-HDL-C and ApoB in ischemic cardiovascular events revealed that ApoB was the most potent indicator of cardiovascular risk (relative risk ratio, 1.43; 95% CI, 1.35 to 1.51). Non-HDL showed an intermediate effect (relative risk ratio, 1.34; 95% CI, 1.24 to 1.44), while LDL-C was the weakest predictor (relative risk ratio, 1.25; 95% CI, 1.18 to 1.33) [58]. Furthermore, ApoB may serve as a superior measure of ASCVD in cases of metabolic syndrome. In contrast to non-HDL-C, ApoB can identify elevated concentrations of small and dense LDL particles (sd-LDL) [59]. Despite the apparent superiority of ApoB, there are limited data comparing ApoB and non-HDL-C as predictors of cardiovascular risk. Further large-scale studies are needed to provide more clarity on this issue.

### 4.4. Autoantibodies against ApoB100: A Novel Biomarker of the Vulnerable Atheromatous Plaque

Mounting evidence from both animal and human studies underscores the association of circulating anti-ApoB100 autoantibodies with cardiovascular burden, presenting them as a potential novel marker for identifying atheromatous vulnerable plaques. Marchini et al. demonstrated elevated plasma levels of pro-inflammatory anti-ApoB IgG in subjects at high cardiometabolic risk, including those with arterial hypertension, obesity, and metabolic syndrome. Conversely, anti-ApoB IgM, suggested to be anti-inflammatory, was notably lower in these patients [60]. High concentrations of IgM antibodies against malondialdehyde (MDA)-modified ApoB100-p210 peptides are believed to correlate with less severe carotid disease in women [61]. On the other hand, Sjögren et al. found that the presence of anti-IgG antibodies against the native peptide 210 of ApoB100 is associated with a reduced risk of CHD [62]. Intriguingly, high levels of p210 ApoB100 antibodies have been linked to a lower incidence of atrial fibrillation in female subjects [63]. These antibodies seem to enhance cholesterol efflux by macrophages. In an experimental model using ApoE-/- mice, Zeng et al. proposed that the increased phagocytosis of oxidized LDL by macrophages is primarily due to the upregulation of Fc receptors, accompanied by increased peroxisome proliferator-activated receptor alpha (PPAR-α) activity and the attenuation of transcription factor NF-κB [64]. Similar results have been demonstrated in humans, revealing a notable inverse association between serum anti-ApoB100 autoantibody levels and plaque burden [65]. 

## 5. ApoB100 in Different Pathological Conditions

### 5.1. Diabetes Mellitus

Increased ApoB100 secretion has been extensively documented in both animal and human models exhibiting insulin resistance, underscoring ApoB100 as an integral component of the metabolic syndrome (MetS). Dyslipidemia observed in type 2 diabetes mellitus (T2DM) is characterized by elevated triglycerides, ApoB, and small dense low-density lipoprotein (sd-LDL) levels, along with a concurrent decrease in HDL-C, all stemming from the underlying insulin resistance. Consequently, insulin is suggested to possess antiatherogenic properties through a combined favorable impact on ApoB production and clearance. Notably, insulin promotes ApoB100 degradation and moderates the secretion of VLDL from the liver [66]. The ApoB/ApoA1 ratio has been identified as associated with T2DM independently, particularly in patients with cardiovascular disease, potentially serving as a predictor of cardiovascular risk in these cases [67].

In individuals with T2DM, there is an increased synthesis of triglycerides by the liver due to the elevated flux of free fatty acids. This results in a heightened production of ApoB and VLDL. Cholesteryl ester transfer protein (CETP) facilitates the exchange of TGs carried by VLDL for cholesteryl esters transported by LDL and HDL. Consequently, levels of atherogenic cholesterol-rich VLDL remnant particles and TG-rich, cholesterol-depleted HDL particles are elevated. Additionally, the formation of sd-LDL originates from the hydrolysis of TG-rich LDL by lipoprotein lipase [68]. The oversecretion of VLDL-ApoB100 may be linked to various mechanisms, including abnormal gene and protein expression, as well as the phosphorylation of different pathways associated with inflammatory insulin signaling. Recent evidence suggests a connection between the ApoB100 rs693 gene polymorphism and metabolic syndrome, indicating a strong association with obesity and increased serum concentrations of total cholesterol (TC), LDL-C, TG, and glucose in these patients [69,70,71].

Individuals with type 2 diabetes mellitus and albuminuria are reported to exhibit increased serum apoB100 levels, suggesting a potential association between ApoB concentrations and the presence of diabetic nephropathy [72]. In a recent study by Taskinen et al., the potential influence of intestinal triglyceride-rich lipoproteins on residual cardiovascular disease risk was investigated in overweight/obese subjects with diabetes mellitus undergoing statin treatment. The results indicated that, compared to the control group, there was no significant difference in the kinetics of ApoB100 in very low-density lipoprotein or in LDL ApoB100 synthesis, pool size, and clearance rate. Contrarily, ApoB48 concentrations were approximately two times higher following a fat-rich meal, with a twofold increase in the density ranges of VLDL1 and VLDL2 [73].

### 5.2. Obesity

Mounting evidence underscores insulin resistance as the primary underlying factor in obesity-associated dyslipidemia, characterized by low serum high-density lipoprotein cholesterol and high triglyceride concentrations. In recent years, attention has turned to the role of pro-inflammatory adipokines, such as resistin. Produced mainly by macrophages, resistin appears to facilitate VLDL synthesis through ApoB100 production, activated by microsomal triglyceride transfer protein (MTP) [74]. Interestingly, Skogsberg et al. have proposed that ApoB100-LDL may serve as a metabolic signal from the liver to peripheral fat, facilitating the inhibition of catecholamine-mediated lipolysis in adipocytes. This dose-dependent effect is believed to be achieved through its binding to the LDL receptor. Additionally, the absence of ApoB100 may result in increased oxygen consumption and lipid oxidation. Therefore, ApoB100 may be identified as a link between atherogenic dyslipidemia and various components of the metabolic syndrome [75]. 

Different levels of treatment, including physical exercise, weight loss, and hypolipidemic agents, have the potential to modify ApoB100 metabolism in patients with metabolic syndrome, including those with ectopic skeletal muscle deposition. Ectopic fat deposition in skeletal muscle is considered a characteristic feature of metabolic syndrome. In insulin-resistant obese individuals, weight loss resulting from a low-fat diet may lead to a significant decrease in visceral and subcutaneous fat areas, liver fat, and skeletal muscle fat, accompanied by a reduction in VLDL-ApoB100 secretion rate. Notably, the percentage decrease in skeletal muscle fat with weight loss is strongly correlated with the corresponding changes in VLDL-ApoB100 concentration (r = 0.770, *p* = 0.009) and VLDL-ApoB100 secretion (r = 0.682, *p* = 0.030) [76].

ApoB100 plays a principal role in peripheral fat deposition, and the correlation between abdominal visceral obesity, low HDL-C levels, and subclinical atherosclerosis has been observed. Postprandial dyslipidemia following a high-fat meal in BMI-matched healthy subjects, as well as in individuals with prediabetes or type 2 diabetes, has been associated with an increase in TGs and ApoB concentrations [77]. Emerging therapeutic agents hold promise for improving lipid metabolism in metabolic syndrome, particularly in individuals with obesity subjected to a high-fat diet. One such agent is Yhhu2407, an inhibitor of diacylglycerol acyltransferase 1 (DGAT1). DGAT1, a membrane-bound O-acyltransferase, plays a crucial role in the synthesis of triacylglycerides, essential for dietary fat absorption and storage. Inhibiting DGAT1 with Yhhu2407 has shown potential in reducing serum TGs following a high-fat meal, as it diminishes free fatty acid-induced TG accumulation and apolipoprotein B100 synthesis in HepG2 cells [78].

### 5.3. Liver Steatosis (NAFLD/MAFLD)

Non-alcoholic or metabolic-associated fatty liver disease (NAFLD/MAFLD) is now recognized as a significant risk factor for cardiovascular disease and is considered a component of the metabolic syndrome. A substantial proportion of individuals with MAFLD are overweight or obese, displaying characteristics such as insulin resistance and atherogenic dyslipidemia. In MAFLD, dyslipidemia is primarily characterized by the chronic deposition of triglycerides in hepatocytes, leading to elevated serum triglyceride levels. Typically, MAFLD patients present with hypertriglyceridemia attributed to large VLDL particles, low HDL-C, and increased levels of small dense low-density lipoprotein (sd-LDL), resembling patterns observed in diabetes mellitus and metabolic syndrome [79]. MAFLD-associated dyslipidemia appears to be most prominent in mild steatohepatitis and progressively diminishes in more advanced stages of liver fibrosis. This dynamic evolution is attributed to alterations in VLDL synthesis and disposal [80,81].

As mentioned earlier, insulin exhibits antiatherogenic features by inhibiting ApoB mRNA translation and inducing ApoB degradation [82,83]. In the context of MAFLD, insulin resistance is linked to increased ApoB expression and delayed ApoB clearance. This association is correlated with triglyceride lipolysis, heightened oxidative stress, and the glycosylation oxidization of lipoprotein particles [84]. ApoB100, as a key component of VLDL molecules, plays a central role in the development of CVD. Interestingly, ApoB100 is strongly associated with NAFLD in individuals with non-genotype 3 hepatitis C. In these cases, higher ApoB100 levels are significantly linked to a greater degree of liver steatosis, attributed to underlying insulin resistance [85].

### 5.4. Polycystic Ovary Syndrome (PCOS)

Polycystic ovary syndrome (PCOS), a common endocrine disorder, is typically linked to insulin resistance and lipid metabolism abnormalities, putting individuals at an increased risk of cardiovascular disease, especially in the presence of obesity. A cross-sectional study conducted in Canada in 2017, focusing on girls aged 12–17 years, reported that adolescent females with PCOS and obesity exhibited elevated fasting and postprandial triglycerides and remnants of ApoB48 and ApoB100 lipoproteins [86]. Combining high doses of fish oil (FO) with metformin appears to have a favorable impact on female patients with PCOS and metabolic syndrome, leading to decreased concentrations of ApoB48 and ApoB100, with beneficial effects on both fasting and postprandial serum TGs and ApoB levels [87]. These findings are clinically significant, suggesting the potential presence of early subclinical cardiovascular risk in these patients [86,87]. In PCOS, the risk of non-alcoholic fatty liver disease is significantly elevated. Androgens are believed to predispose to liver steatosis through mechanisms that are not yet fully understood. An experimental model in female Sprague Dawley rats by Wang et al. showed that hyperandrogenemia (HA) may lead to liver triglyceride accumulation via the downregulation of microsomal triglyceride transfer protein expression [88]. Additionally, estrogens may contribute to abnormal lipid metabolism, as evidenced by their promotion of the production of ApoB100-containing lipoproteins by human placental BeWo cells [89].

### 5.5. Peripheral Artery Disease (PAD)

The lipid profile, particularly lipoprotein parameters, has not been extensively studied in patients with peripheral artery disease compared to other cardiovascular diseases. In a population study by Bertoia et al., the impact of oxidized phospholipids (Ox-PL) on apolipoprotein B100-containing lipoproteins (Ox-PL/ApoB) was investigated in relation to the risk of PAD. Ox-PL/ApoB levels were found to be positively associated with PAD risk, irrespective of gender. Notably, autoantibodies to apolipoprotein B100 immune complexes (ApoB-IC) did not consistently show a relationship with PAD risk [90]. More recently, the SURDIAGENE study revealed independent associations between anti-atherogenic molecules, such as HDL-C and apolipoprotein A1, and the incidence of major PAD in individuals with T2DM. Elevated non-HDL-C was also linked to an increased prevalence of severe PAD. However, no significant correlation was found between ApoB100 and severe PAD [91]. These findings highlight the importance of specific lipid parameters, such as Ox-PL/ApoB and non-HDL-C, in assessing the risk and severity of PAD. The variability in the associations between different lipoprotein components and PAD emphasizes the need for further research to better understand the lipid profile’s role in the development and progression of peripheral artery disease.

### 5.6. Chronic Kidney Disease (CKD)

Among patients with atherosclerotic cardiovascular disease, those with chronic kidney disease (CKD) exhibit a distinctive lipid profile characterized by prevalent hypertriglyceridemia and low levels of HDL-C. LDL-C typically results low in subjects with renal failure, and its levels are even lower in patients on hemodialysis [92]. In comparison to patients without CKD, those with CKD often present with more diffuse atheroma plaque and a higher burden of calcification. An aberrant metabolism of apolipoprotein B-containing lipoproteins and their remnants is reported in CKD, emphasizing the potential significance of monitoring apolipoprotein B levels in assessing cardiovascular mortality and morbidity, especially in end-stage renal disease (ESRD). Elevated levels of lipoprotein(a) may also be observed, and a high ApoB/ApoA1 ratio is likely associated with an increased cardiovascular risk [93].

High ApoB levels may contribute to the dysregulation of glomerular endothelial cells and vessels, potentially accelerating CKD progression due to inflammation and oxidative stress [94]. In CKD, elevated levels of ApoB-containing particles are thought to result from defective receptor removal combined with a reduction in enzymatic delipidation [95]. In a cross-sectional study involving 146,533 participants, Xu reported that ApoB showed the strongest association with CKD among all lipid parameters. Moreover, elevated serum ApoB values may potentially precede the clinical manifestation of CKD [96]. These insights underscore the importance of monitoring ApoB and other lipid parameters in assessing cardiovascular risk and kidney disease progression in individuals with CKD.

## 6. ApoB Gene Mutations and Cardiovascular Risk

### 6.1. Familial Defective Apolipoprotein B100 (FDB)

Familial defective apolipoprotein B100 (FDB) is an inherited autosomal codominant disorder characterized by hypercholesterolemia and premature atherosclerosis. The molecular defect in FDB involves a point mutation leading to the substitution of glutamine for arginine at amino acid position 3500 (R3500Q) of ApoB100. This results in an aberrant binding affinity of ApoB100 to the LDL receptor, lowering LDL clearance and enhancing LDL accumulation in the plasma [97]. The R3500W mutation, a tryptophan-for-arginine substitution at the same codon, is the second most common related mutation in FDB. The R3500Q variant has been shown to increase serum LDL-C by 60–70 mg/dL [98]. Familial defective apolipoprotein B100 has been linked to increased rates of both mild and severe coronary artery calcification, and patients often experience early cardiovascular events, including coronary artery disease, ischemic heart disease, and myocardial infarction [98,99]. Both APOB R3500Q and R3500W variants are also implicated in familial hypercholesterolemia (FH), accounting for 12% of FH cases. In fact, about 35 FH-causing variants due to ApoB have been described [100]. However, pathogenic mutations in APOB tend to be less severe than LDL-R mutations, which are primarily involved in FH pathogenesis, leading to milder elevations in serum LDL-C [101].

Vogt et al. demonstrated that early cardiovascular disease is more common in FH compared to FDB. Almost 40% of FH patients had coronary heart disease (CHD) at a mean age of 41 years, while CHD was present in 5.6% of FDB subjects with a median age of 52 years. Total cholesterol and LDL-C levels were significantly higher in FH than in FDB men. Additionally, internal carotid artery stenoses were more prevalent in FH (15% versus 4% in the FDB group). However, the frequency of peripheral artery disease and hypertension was nearly identical between the two groups [102]. Distinguishing between FDB and FH may have treatment implications, as certain lipid-modifying agents may act via the LDL-R, which is typically normal in subjects with FDB.

### 6.2. ApoB-Related Familial Hypobetalipoproteinemia (FHBL)

Familial hypobetalipoproteinemia (FHBL) is an autosomal codominant genetic disorder characterized by the diminished secretion of ApoB48 and ApoB100 lipoproteins due to defects in the ApoB gene. In these cases, mutations involve single nucleotide deletions or substitutions leading to truncated protein production through premature translation termination. Over 60 mutations causing truncations in the ApoB gene have been identified as contributors to FHBL. Less frequently, FHBL can result from loss-of-function mutations in the PCSK9 gene. The clinical presentation of FHBL varies, with heterozygous APOB-FHBL individuals usually being asymptomatic or exhibiting mild liver steatosis, often requiring no treatment. However, in 5–10% of cases, more severe steatohepatitis may develop, potentially progressing to liver cirrhosis [103].

Biallelic or homozygous familial hypobetalipoproteinemia presents with a broad spectrum of clinical findings, including steatorrhea due to fat-soluble vitamin deficiency, gastrointestinal and neurological symptoms. Patients commonly exhibit hepatomegaly, growth deficiency, and are susceptible to fatty liver disease. Notably, despite triglyceride accumulation in hepatocytes, the incidence of insulin resistance and diabetes mellitus is not increased. Laboratory tests typically reveal abnormal serum aminotransferases and severe hypocholesterolemia, with very low or absent LDL-C, triglycerides, and ApoB concentrations. Acanthocytosis is a pathognomonic laboratory feature [104,105]. If left untreated, patients may experience neurological symptoms such as tremors, dysarthria, ataxia, and muscle pain or weakness. Atypical pigmentation of the retina may also be observed. Diagnosis is based on molecular genetic testing, and management is typically supportive. Patients are advised to follow a low-fat diet, constituting less than 30% of total calories, along with high-dose oral fat-soluble vitamin supplementation. During pregnancy, vitamin A administration should be reduced by 50% as it may be harmful to fetal development [106].

The correlation between FHBL and reduced cardiovascular risk remains unclear, although most available evidence suggests that individuals with FHBL may be protected from cardiovascular disease. Sankatsing et al. demonstrated that FHBL patients face an increased risk of hepatic steatosis but a decreased risk of cardiovascular disease, as indicated by reduced carotid arterial stiffness [107]. Additionally, Peloso et al. reported that rare protein-truncating mutations in APOB, leading to lower LDL-C and triglyceride levels, could potentially offer protection against CHD [108]. In a meta-analysis comprising 12 case–control studies with 57,973 subjects, including 18,442 with early-onset CHD, the presence of an ApoB truncation was associated with a 72% reduction in coronary heart disease (odds ratio, 0.28; 95% confidence interval, 0.12–0.64; *p* = 0.002) [109].

## 7. The Effects of Lipid-Modifying Interventions on ApoB-100

### 7.1. Lifestyle Interventions

Lifestyle interventions play a crucial role in reducing the burden of cardiovascular disease, involving modifications in both diet and exercise. The Mediterranean diet has demonstrated favorable outcomes in reducing LDL-C levels, accompanied by a decrease in ApoB100-LDL concentrations. This effect is believed to be achieved through enhanced LDL catabolism, irrespective of weight loss, particularly in men with metabolic syndrome. In these cases, the impact of the Mediterranean diet on very low-density lipoprotein levels and kinetics seems to be neutral unless significant weight loss is observed [110]. In contrast, the Western diet, characterized by the increased consumption of dietary saturated fatty acids, has unfavorable effects by impeding the clearance of LDL ApoB100 from circulation [111]. 

Beyond dietary modifications, aerobic exercise training (AET) is proposed to have a positive impact on the standard lipid profile, while simultaneously attenuating CVD risk. A multivariate meta-analysis of 57 randomized controlled trials involving 3194 participants revealed that AET can particularly exert a beneficial impact on apolipoproteins and lipoprotein ratios. Moreover, CVD risk predicted by these biomarkers may be reduced when AET is employed as both a preventive and treatment intervention. Specifically, AET at >40% VO2MAX for ≥12 weeks significantly increased anti-atherogenic and lipoprotein sub-fractions while also elevating atherogenic lipid ratios. AET beneficially lowered ApoB100 by 2.073 mg/dL, suggesting a potential 4.34% reduction in CHD risk, given that a 9% decrease in CHD risk occurs for every reduction in ApoB100 [112,113].

### 7.2. Traditional Hypolipidemic Agents

#### 7.2.1. Statins

Statins are the cornerstone of hypolipidemic treatment, with a unique mechanism of action involving HMG-CoA reductase inhibition. The primary effect of their administration is the decrease in ApoB-containing lipoproteins, especially LDLs, via hepatic reduction in ApoB synthesis or the enhancement of LDL-R clearance [114]. Twenty years ago, Watts et al. investigated the effects of atorvastatin on liver ApoB100 production in obese men with metabolic syndrome and showed that atorvastatin attenuates the synthesis of cholesterol. On the contrary, gut cholesterol absorption was increased, suggesting that this elevation may counterbalance the inhibitory impact on hepatic VLDL-ApoB secretion without interfering with enhanced VLDL-ApoB catabolism [115]. A randomized, double-blind, placebo-controlled trial involving nine individuals with hypercholesterolemia and hypertriglyceridemia investigated the influence of different doses of atorvastatin on human ApoB100 kinetics in triglyceride-rich lipoprotein (TRL), IDL, and LDL. Compared with placebo, atorvastatin at 20 mg per day promoted a significant decrease in TLR, IDL, and LDL ApoB-100 pool size, as a consequence of the enhanced fractional catabolic rate (FCR). Notably, no significant alterations in production rate (PR) were present. The daily use of 80 mg atorvastatin seems to boost this effect, indicating that atorvastatin has a dose-dependent impact on LDL ApoB100 kinetics [116].

In 2006, the SPARCL trial showed that in individuals with recent stroke or TIA, without concomitant coronary heart disease, aggressive LDL-C lowering with 80 mg of atorvastatin limited both the overall incidence of stroke and major cardiovascular events [117]. Based on these results, the SPARCL trial has shown that oxidized phospholipids associated with ApoB100-containing lipoproteins (Ox-PL-ApoB) may predict the recurrence of stroke and the incidence of coronary events over a 5-year period. This biomarker is believed to reflect Lp(a) levels since lipoprotein(a) is a major vehicle of oxidized phospholipids. Notably, the study reported that the inhibition of Ox-PL-ApoB was independent of the dose regimen, suggesting that these favorable effects of statins may be mainly achieved via non-Ox-PL pathways [118]. In disagreement with the aforementioned results, Chemello et al. pointed out that Ox-PL-ApoB levels measured in SPARCL subjects were higher among diabetic patients, while Lp(a) levels are expected to be decreased in these cases. Furthermore, Ox-PL-ApoB concentrations were markedly reduced during the follow-up period, although Lp(a) concentrations are likely to increase in response to high-intensity statin therapy [119].

Non-high-intensity statins, such as pravastatin and pitavastatin, have also demonstrated favorable effects on serum ApoB100 levels. In individuals with non-insulin-dependent diabetes, pravastatin has been reported to lead to a 26.6% decrease in ApoB100 levels over a 4-year prescription period [120]. Similarly, pitavastatin administration at 4 and 8 weeks has shown benefits in ApoB levels and the ApoB/ApoA1 ratio, with more favorable effects observed in combination treatment with ezetimibe [121]. Furthermore, the maximal dose of rosuvastatin at 40 mg/day appears to increase the catabolism of LDL- ApoB100 without affecting ApoB100 PR in a dose-related manner [122]. In the STELLAR clinical trial, rosuvastatin at doses ranging from 10 mg to 40 mg was reported to be superior compared to other statins in improving the lipid profile in subjects with hypercholesterolemia, including ApoB levels. Specifically, rosuvastatin reduced ApoB by 36.7% to 45.3%, in contrast to 29.4% to 42.9% with atorvastatin, 22.2% to 34.7% with simvastatin, and 14.7% to 23.0% with pravastatin [123].

Recently, the STAtins Reduce Thrombophilia trial evaluated the role of rosuvastatin in reducing coagulation factor values via mutual mechanisms of synthesis or regulatory pathways with apolipoproteins. The authors concluded that in individuals with prior venous thrombosis, the use of rosuvastatin resulted in the reduction of the concentrations of several lipoproteins, but this decrease was correlated only with a decrease in FVII and FXI serum levels. Among different lipoproteins, ApoB100 showed the highest mean decrease of −0.43 g/L, and this reduction concerned exclusively the FXI levels [124].

#### 7.2.2. Ezetimibe

Ezetimibe is an azetidine derivative that selectively blocks Niemann-Pick C1-like protein (NPC1L1), resulting in the inhibition of intestinal cholesterol absorption. In addition, the incorporation of cholesterol into chylomicrons is decreased, leading to an incremental reduction in the intrahepatic cholesterol pool. Thus, LDL-R expression is enhanced, favoring the removal of ApoB100-containing lipoproteins from the plasma. When used as monotherapy, ezetimibe reduces LDL-C by 15–22%, as attenuated cholesterol absorption leads to a compensatory increase in its synthesis [125]. On the other hand, ezetimibe co-administration with statins seems to promote a clear-cut synergic effect, resulting in an additional 15% to 25% reduction in serum LDL-C, regardless of the statin type and dose [126].

Tremblay et al. evaluated the effects of ezetimibe on the in vivo kinetics of ApoB48 and ApoB100 in humans and found that treatment with ezetimibe reduced serum LDL-C levels due to an improvement in the fractional catabolic rate (FCR) of ApoB100-containing lipoproteins. In particular, the LDL-ApoB100 pool size (PS) was markedly diminished by 23.2%, as a result of a significant 24% increase in the FCR of this lipoprotein fraction. Furthermore, IDL- and VLDL-ApoB100 FCRs were elevated by 20.8% and 31.2%, respectively, while an increased VLDL-ApoB100 production rate (PR) was observed [127]. In another study, the same author evaluated the addition of 40 mg simvastatin to ezetimibe, reporting a further decrease in LDL-C levels. This reduction is thought to be mediated by an increase in FCR of the ApoB100-containing lipoproteins. Overall, ezetimibe alone leads to a reduction in ApoB by 19.8%, whereas combination treatment with simvastatin results in a significant decrease in ApoB by 47.4% [128]. In fact, today, the main consensus documents suggest combination therapy with a high-intensity statin along with ezetimibe as the first-line treatment in individuals with very high or extremely high CVD risk [129].

Using a knockout (LDLR-/-) Syrian golden hamster, Lin et al. have shown that a high-cholesterol, high-fat diet (HFD) results in atherosclerosis, as it may increase serum TGs, ApoB48, and ApoB100 levels. The authors have hypothesized that triglyceride-rich lipoproteins and their remnants stimulate inflammation pathways, resulting in impaired endothelial function, which in turn causes the infiltration of the arterial wall by macrophages. Notably, the use of ezetimibe for 14 days reduced these large particles but not LDL-C [130].

#### 7.2.3. Fibrates

Fibrates are a type of amphipathic carboxylic acids, mainly used for the treatment of severe hypertriglyceridemia, due to their ability to stimulate nuclear peroxisome proliferators-activated receptor-α (PPAR-α). Their use results in the reduction of serum triglycerides by approximately 50%. Secondarily, fibrates increase HDL-C by 5–15%, whereas their impact on LDL-C is limited, as they decrease serum LDL-C only by 10–25% [131,132]. 

Fibrates are thought to promote the reduction of VLDL-ApoB100 by a combined effect on its fractional catabolism and production. Nine studies have investigated the impact of PPAR-agonism on apolipoprotein kinetics in patients with different lipoprotein phenotypes, evaluating the effects on VLDL-ApoB100 clearance, determined as the fractional catabolic rate (FCR). All studies demonstrated an elevation in the VLDL-ApoB100 FCR, ranging from 33 to 325%. Of note, the greatest impact (101–325%) was observed in subjects with triglyceride levels higher than 500 mg/dL, except for patients with familial dysbetalipoproteinemia [133]. On the contrary, LDL-ApoB100 levels decrease exclusively as a result of elevated LDL fractional catabolism, with the exception of individuals with triglyceride concentrations exceeding 400 mg/dL. In such cases, LDL-ApoB FCR, in response to fibrate use, seems to be reduced, likely due to the activation of the reticuloendothelial system. As a result, increased LDL-ApoB100 uptake by scavenger macrophage receptors within the reticuloendothelial system may be seen [133,134]. Alternatively, high TG levels may provoke oxidative damage in the ApoB-containing lipoproteins, leading to enhanced uptake by macrophages [135].

Fenofibrate, the most commonly used fibrate, has been shown to reduce plasma ceramide, independently of the usual lipid parameters, in subjects with diabetes mellitus type 2. Ceramides are biologically active sphingolipids that play a key role in the development of atherosclerosis, as they highly accumulate in the atheromatous plaque. In this context, ceramides are suggested as potential novel predictors of ASCVD. Interestingly, the administration of fenofibrate seems to reduce the concentrations of TGs and LDL-C, with a concomitant decrease in ApoB100 levels (−27.0%, *p* < 0.01) in these cases [136]. This finding is of high importance, as the favorable effects of fenofibrate on cardiovascular outcomes are still conflicting.

#### 7.2.4. Omega-3 Fatty Acids (FAs)

Omega-3 fatty acids are considered a supplemental intervention in individuals with hypertriglyceridemia. Their use leads to the intrahepatic degradation of ApoB48 and ApoB100, although they stimulate cellular TG production. Their impact is greater on ApoB100 compared to ApoB48 and seems to be associated with the percentage of ApoB lipidation [137]. Omega-3 fatty acids seem to provide beneficial effects in the metabolism of ApoB100-containing lipoproteins in subjects with diabetes mellitus type 2. In these cases, n-3 PUFAs lead to the suppression of VLDL production, as well as its conversion into LDL-C, without influencing serum cholesterol levels [138]. Thomas et al. have shown that in ApoB100 LDLr-/- mice, ACAT2 enhances dietary fat type-specific atherosclerosis, as its removal by gene deletion limited atherogenic plaque formation. ACAT2 has a key role in the hepatic production of CE, an atherogenic compound of ApoB-containing lipoproteins [139].

### 7.3. Lipoprotein Apheresis (LA)

Lipoprotein apheresis (LA) is recommended for patients with severe hypercholesterolemia who do not attain therapeutic goals through lifestyle and pharmacologic interventions, including those with FH and LDL-C levels exceeding 300 mg/dL. LA specifically eliminates ApoB100-containing lipoproteins, leading to a significant reduction in apolipoprotein B concentrations, typically ranging from 51% to 69%. Additionally, LA exhibits pleiotropic properties in atherosclerosis, by mitigating inflammation and thrombosis. Various techniques are available, and LA is generally considered well tolerated. This modality results in a decrease in LDL-C, Lp(a), and TGs by approximately 50–75% and 50%, respectively [140]. Yaroustovsky et al. reported statistically significant reductions in LDL-C and Lp(a) levels, using two different LA methods (H.E.L.P.-apheresis and cascade lipid-filtration). Both groups demonstrated a positive trend in the ApoB100/ApoA ratio (with a decrease of 33–60%) and a reduction in the atherogenic index (38% and 53%, respectively). Notably, ApoB100 levels were decreased by 54% in the H.E.L.P.-apheresis group and by 70% in the cascade lipid-filtration group [141]. However, a notable drawback of this method is the rebound phenomenon observed, as LDL-C and Lp(a) concentrations return to baseline levels within almost two weeks after its utilization [140]. Furthermore, the impact of this method on cardiovascular burden remains unclear. Nevertheless, numerous studies, primarily focusing on Lp(a), support the notion that LA may decrease elevated Lp(a) levels and major CVD events by 53–74% and 54–90%, respectively [6]. These findings have been reinforced by preliminary data from the German Lipoprotein Apheresis Registry (GLAR), where Lp(a) levels were lowered by 72%, and major CVD events were reduced by 90% [142].

### 7.4. The New Era of Lipid-Modifying Therapies

#### 7.4.1. Mipomersen and Lomitapide

Mipomersen and lomitapide are lipid-lowering agents mainly used in patients with homozygous familial hypercholesterolemia (HoFH). Mipomersen, a second-generation antisense oligonucleotide inhibitor of ApoB100, facilitates the selective degradation of ApoB100 mRNA, independent of LDL-R function. Consequently, it impairs the production of ApoB100-enriched lipoproteins, leading to reduced levels of LDL-C, VLDL-C, and Lp(a) synthesis. Previous clinical evidence has demonstrated that mipomersen induces a dose-dependent reduction in LDL-C in patients with hypercholesterolemia, including those with familial hypercholesterolemia [143]. In a randomized, double-blind, placebo-controlled, multicenter trial (enrolling 39 subjects in the mipomersen arm vs. 19 in the placebo arm) with LDL-C ≥ 301.16 mg/dL, mipomersen administration resulted in a significant 36% reduction in ApoB from baseline [144]. Similar results were reported in another study, involving patients receiving weekly subcutaneous doses of 200 mg for 26 weeks. In comparison to placebo, mipomersen significantly reduced ApoB by 26.3% [145]. Although all available studies affirm the efficacy of mipomersen in reducing ApoB levels compared to placebo, concerns about side effects, particularly hepatotoxicity, have been reported following mipomersen use [143].

On the other hand, lomitapide is an oral selective inhibitor of microsomal triglyceride transfer protein (MTP), which impacts the production of VLDL lipoproteins primarily containing ApoB100. In comparison to mipomersen, lomitapide is believed to exert more potent effects on the lipid profile. Specifically, while mipomersen is associated with an LDL-C reduction of approximately 25%, lomitapide is thought to induce a more substantial decrease of around 50% [146]. Presently, lomitapide is indicated for individuals with homozygous familial hypercholesterolemia (HoFH). Regarding ApoB levels, lomitapide appears to reduce the production rate of LDL ApoB by nearly 70% [147]. The primary side effects of lomitapide include diarrhea and hepatotoxicity. Similar to mipomersen, lomitapide may predispose individuals to liver steatosis, necessitating the close monitoring of patients [147,148].

#### 7.4.2. Bempedoic Acid (BA)

Novel lipid-lowering therapies are emerging, aiming at reducing the risk of cardiovascular disease, particularly in subjects with refractory hypercholesterolemia or statin intolerance. Bempedoic acid (BA) is a novel class of non-statin LDL-lowering intervention with a distinctive mechanism of action, involving the downstream of cholesterol synthesis, accompanied by an upregulation of the LDL-R [149]. BA’s impact on LDL-C levels depends on prior statin use, whereas BA contributes significantly to the reduction of LDL-C concentrations among statin-intolerant individuals. Bempedoic acid’s safety and efficacy in patients with statin intolerance have been investigated in phase 3 CLEAR trials, showing that BA may promote a 12–19% reduction in ApoB100 [150]. CLEAR Tranquility (NCT03001076) is a population study consisting of 269 patients with statin-intolerant hypercholesterolemia and LDL-C ≥ 100 mg/dL, thus requiring additional LDL-C lowering. The aim of this study was to investigate the safety and efficacy of BA when added to a prior lipid-modifying treatment that included ezetimibe. The use of bempedoic acid resulted in the reduction of LDL-C by 28.5% compared to placebo. Regarding secondary endpoints, BA decreased ApoB by 19.3%, being well tolerated, as BA-related side effects were similar between the BA and placebo group [151].

#### 7.4.3. PCSK9 Inhibition

PCSK9 is a serine protease that facilitates the degradation of the LDL-R. Variants in the PCSK9 gene are thought to be implicated in the expression of this receptor. In particular, loss-of-function mutations attenuate PCSK9′s activity and induce LDL-R activity, resulting in low LDL-C levels and reduced cardiovascular risk. On the other hand, gain-of-function mutations exert the opposite effects, producing a phenotype similar to familial hypercholesterolemia (FH). Thus, agents that inhibit PCSK9′s activity may be effective in reducing serum cholesterol levels [152]. Currently, alirocumab and evolocumab are the two available, fully human immunoglobulin G subtype antibodies against PCSK9. They bind with a 1 to 1 ratio to serum PCSK9, inhibiting PCSK9′s affinity to bind to the LDL-R. The intrahepatic accumulation of the LDL-R favors the clearance of the LDL-C particles, leading to their reduction [153]. 

PCSK9 inhibitors may also be beneficial in the treatment of postprandial hypertriglyceridemia. Existing evidence supports that PCSK9 may provoke intestinal triglyceride-rich-ApoB production, although the underlying mechanisms are still unclear. Rashid et al. have shown that the exposure of human enterocytes to 10 μg/mL of recombinant human PCSK9 for 24 h enhanced ApoB100 synthesis by 55%, while the short-term inhibition of PCSK9 counterbalanced this effect. ApoB activation has been ascribed to its increased stability and mRNA expression, via both LDL-R-dependent and -independent mechanisms. This is thought to be attributed to transcriptional effects on lipogenic genes, MTP, and ApoB, and to posttranscriptional features on MTP and LDL-R, respectively [154]. PCSK9 and diabetes mellitus are significant predisposing factors regarding LDL-C catabolism. Interestingly, Verges et al. underscore that the impact of diabetes mellitus on LDL-ApoB catabolism may overshadow the effects of PCSK9, as serum PCSK9 seems to affect LDL-ApoB-100 catabolism only in individuals without T2DM [155]. Furthermore, PCSK9 inhibitors seem to lower Lp(a) levels, as shown in several clinical trials. Croyal et al. have pointed out that alirocumab may reduce serum Lp(a), as the drug facilitated ApoB catabolism and diminished Apo(a) production [156].

Inclisiran is a novel small interfering ribonucleic acid (siRNA) therapeutic agent that inhibits hepatic PCSK9 production, thus alleviating the PCSK9-mediated degradation of the LDL receptors. As a consequence, greater LDL-R expression and LDL-C uptake by the liver are observed, leading to the reduction in serum LDL-C concentrations. The ORION-10 and the ORION-11 phase 3 trials evaluated the efficacy and the safety of inclisiran in high-CVD-risk patients with increased LDL-C levels. Both trials showed that inclisiran reduced LDL-C levels by approximately 50%. In addition, inclisiran showed a favorable profile in all atherogenic lipoproteins, including ApoB [157].

#### 7.4.4. Angiopoietin-Like 3 Protein (ANGPTL3) Inhibitors

Recently, the Food and Drug Administration (FDA) granted approval for a new class of lipid-lowering agents known as angiopoietin-like 3 protein (ANGPTL3) inhibitors. The inhibition of ANGPTL3 is associated with a heightened activity of lipoprotein lipase (LPL) and endothelial lipase (EL). In turn, LPL and EL enhance the clearance of VLDL remnants through liver receptors, favoring a reduction in LDL-C levels, irrespective of LDL-R activity. The primary indications for their use are refractory homozygous familial hypercholesterolemia and mixed dyslipidemia. At present, evinacumab and vupanorsen stand as the primary ANGPTL3 inhibitors in use [158]. 

Evinacumab, a monoclonal antibody binding to ANGPTL3, leads to a reduction in LDL-C primarily by enhancing the clearance of ApoB-containing lipoproteins from circulation. Administered at a dose of 20 mg/kg per month, evinacumab results in a 25% decrease in LDL-C, a 31% decrease in ApoB, and a 46% reduction in non-HDL-C [159]. A study by Rosenson et al. investigated the long-term safety and efficacy of this agent in subjects with refractory hypercholesterolemia. Its prescription showed a sustained reduction in LDL-C concentrations without significant side effects. A 72-week follow-up revealed a mean decrease in LDL-C by 45.5%, in ApoB by 38.8%, in non-HDL-C by 48.4%, and in fasting triglycerides by 57.2% [160]. On the other hand, vupanorsen is an antisense oligonucleotide (ASO)-targeting hepatic ANGPTL3 mRNA for degradation. In comparison to a placebo, vupanorsen appears to have a modest impact on lipid parameters in individuals with type 2 diabetes mellitus, fasting hypertriglyceridemia, and liver steatosis. It has been reported to lower LDL-C, ApoB, and non-HDL-C by up to 18%, 12%, and 9%, respectively [159].

#### 7.4.5. Cholesteryl Ester Transfer Protein (CETP) Inhibitors

Cholesteryl ester transfer protein (CETP) is a liver glycoprotein responsible for the exchange of triglycerides (TGs) from VLDL particles with cholesteryl esters from LDL and HDL particles. This interplay yields VLDL and LDL particles with cholesteryl esters and HDL particles with TGs, whereas VLDL and LDL molecules are depleted of TGs, and HDL particles of cholesteryl esters. Thus, CETP inhibitors are the first drugs that seek to significantly increase HDL-C. Hitherto, none of the CETP inhibitors has ever achieved marketing authorization since all of them failed to minimize ASCVD risk in randomized controlled trials (RCTs) [161].

Current evidence regarding the novel CETP inhibitor, obicetrapib, is promising as it has demonstrated the ability to lower LDL-C and ApoB without leading to major side effects. In a phase 2 clinical trial involving 364 patients with mild dyslipidemia (TULIP), the prescription of obicetrapib at doses ranging from 1 to 10 mg for 12 weeks, both alone and in combination with moderate-intensity statins, showed favorable outcomes in ApoB concentrations. Specifically, ApoB was significantly reduced by 20.0%, 24.6%, 33.6%, and 33.7% with the 1, 2.5, 5, and 10 mg obicetrapib doses, respectively. Additionally, there was a reduction of 50.1% and 46.3% when obicetrapib was combined with atorvastatin and rosuvastatin, respectively [162]. In the Randomized Study of Obicetrapib as an Adjunct to Statin Therapy (ROSE), obicetrapib resulted in a median reduction of LDL-C by approximately 43–50%, along with a decrease in ApoB by 24.4–29.8%, and non-HDL-C by 38.9–44.4% [163]. Presently, obicetrapib is undergoing investigation in PREVAIL, a cardiovascular outcome trial expected to be completed in 2026 [161].

Atherosclerosis is a chronic and progressive inflammatory process that plays a central role in the development of cardiovascular disease. Statins remain the cornerstone of lipid-lowering treatment; however, numerous hypolipidemic agents with diverse mechanisms of action have demonstrated beneficial effects on both LDL-C and ApoB levels. These agents are particularly utilized as adjunctive therapy in cases of refractory hypercholesterolemia or statin intolerance. The major effects of lipid-lowering therapy on ApoB are detailed in Table 1. 

## 8. Immunomodulation of ApoB100 in the Treatment of Atherosclerosis

Inflammation is the primary modulator of atherosclerosis, contributing significantly to the initiation, maintenance, and progression of the atherosclerotic process. Thus, the development of agents that alleviate the activation of the inflammatory cascade may be a novel, promising perspective in the reduction of cardiovascular risk. In this field, NLRP3 inflammasome inhibition, and as a result, the diminished production of IL-1β and IL-1α, may be an attractive novel approach. Colchicine, a traditional NLRP3 inhibitor, has been reported to be beneficial in reducing cardiovascular events in individuals with coronary artery disease (CAD), as shown in the Colchicine Cardiovascular Outcomes Trial and the Low-Dose Colchicine for Secondary Prevention of Cardiovascular Disease Trial [164,165]. A decrease in major adverse cardiovascular events has also been demonstrated following the use of anti-IL-1β or anti-IL-1α antibodies. Combined IL-1β and IL-1α antagonism, via the use of Anakinra, in individuals with ST-segment elevation myocardial infarction (STEMI), may decrease the incidence of death, as well as the hospitalization and the prevalence of new-onset heart failure. Moreover, the injection of a specific immunoglobulin G1 monoclonal antibody following superficial femoral artery angioplasty may result in a decreased incidence of restenosis and major adverse cardiovascular events [166,167].

In recent years, it has become evident that vascular inflammation is influenced by an intricate network of autoimmune responses directed against modified self-antigens within the atherosclerotic plaque. Current experimental evidence reveals that Th1 cells contribute to the progression of atherosclerosis, while regulatory T cells exert a protective role. The prevailing notion suggests that the presentation of modified self-antigens in the inflammatory milieu of atherosclerotic plaques tilts the balance towards the generation of antigen-specific Th1 cells, leading to a localized breakdown of tolerance, while regulatory T cells are diminished. This conceptual framework has prompted the exploration of plaque-antigen tolerogenic vaccines aimed at mitigating plaque inflammation and impeding disease advancement [168]. 

In contemporary research, ApoB100 has emerged as the primary target of immune responses against oxidized-LDL particles, given that LDL oxidation can result in ApoB100 disintegration into smaller fragments. Many of these fragments undergo modification through malondialdehyde (MDA) binding, leading to the production of anti-ApoB100 antibodies. Antibodies targeting ApoB100 may recognize specific epitopes, inducing a distinct peptide configuration identified as a damaged self-antigen. Peptides such as p210 show promise in diminishing the development of atheromatous plaques when utilized as prototype antigens in vaccine formulations. Consequently, p210-based immunization is proposed to limit plaque burden, primarily through a p210-specific CD8+ T cell population [169]. Vaccination against T-cell epitopes of native ApoB100 may alleviate vascular inflammation by macrophages and reduce atheromatous plaque size [170]. Interestingly, the intranasal administration of an ApoB100 peptide fused to the B subunit of cholera toxin (CTB) over a 12-week period has demonstrated a 35% reduction in aortic lesion size. This reduction is accompanied by the induction of type 1 regulatory cells that inhibit T effector responses to ApoB100 [171]. 

Recent experimental findings have elucidated the potential of ApoB100 as a novel therapeutic target against obesity induced by a high-fat diet (HFD). Consequently, an ApoB100 mimotope, peptide pB1, may prevent obesity by recognizing native ApoB100 through pB1-reactive antibodies. Anti-pB1 antibodies enhance lipolysis, diminish LDL uptake from adipocytes, and simultaneously promote LDL uptake by macrophages [172]. As a result, an ApoB100-mimetic vaccine is suggested to prevent obesity and hepatic steatosis in ApoE-/- rats without affecting atherosclerotic plaque formation [173].

The activation of the inflammatory cascade has also been considered to be involved in the increased CVD risk in patients with liver disease. In fact, liver steatosis may promote pro-inflammatory T-cell responses against ApoB100, which acts as an autoantigen recognized by atherogenic CD4+ T cells in subjects with CVD. In such cases, the expression of the pro-inflammatory cytokine interleukin (IL)-17A seems to be raised. In addition, the expression of the regulatory T (Treg)-cell transcription factor FOXP3 may be decreased, predisposing immune dysregulation, and enhanced chronic inflammation [174]. Recently, Wang et al. have shown that the knockdown of lncRP11-675F6.3 may result in a significant decrease in microsomal triglyceride transfer protein and ApoB100 in hepatocytes, accompanied by enhanced intracellular triglyceride levels and autophagy. In turn, accelerated triglyceride accumulation likely associated with autophagy promotes ApoB100 degradation into the autophagosomes, thereby alleviating VLDL assembly. lncRP11-675F6.3 is a noncoding RNA (lncRNA) upregulated in response to the mTOR signaling pathway and is thought to limit high-fat-diet-induced NAFLD by modulating VLDL-associated proteins and autophagy [175].

## 9. Conclusions

Apolipoprotein B is the main component of all major atherogenic lipoproteins. The retention of the ApoB-containing lipoproteins in the vascular subendothelium is fundamental in the induction and development of atherosclerosis. As there is only one ApoB molecule in each ApoB-containing lipoprotein particle, the measurement of ApoB provides a measure of particle number. Therefore, ApoB may be superior to LDL-C in the prediction of cardiovascular risk, particularly in subjects with residual risk. Residual risk is thought to be a result of the underlying inflammatory atherosclerotic process. Lipid-lowering treatment remains the conventional intervention in both the primary and secondary prevention of CVD. Nevertheless, the elucidation of the involved mechanisms regarding inflammation may yield novel therapeutic horizons in the reduction of cardiovascular burden. 

## Figures and Tables

**Table 1:** Main proposed mechanisms regarding the effects of lipid-modifying therapy on apolipoprotein B (ApoB).

Lipid-Lowering Agent	Mechanism of Action	Proposed Mechanisms Regarding the Effects on ApoB
**Statins** [115,116,117,122]	HMG-CoA reductase inhibitor	1.↓ the entry of ApoB-containing lipoproteins in plasma 2.↑ FCR of ApoB-containing lipoproteins 3.(-) PR of ApoB-containing lipoproteins
**Ezetimibe** [127,128,130]	Selective blockade of NPC1L1	1.↑ FCR of ApoB-containing lipoproteins 2.↓ PR of ApoB-containing lipoproteins 3.↓ expression of vascular adhesion factors in endothelial cells 4.↓ lipid uptake by macrophages
**Fibrates** [133,134,135]	PPAR-α agonism	1.↑ FCR of VLDL-ApoB100 2.↓ PR of VLDL-ApoB100 3.↑ FCR of LDL-ApoB100 in TG < 400 mg/dL 4.↓ FCR of LDL-ApoB100 in TG > 400 mg/dL due to activation of RES: - ↑ uptake LDL-ApoB100 by macrophages due to ↑ uptake by scavenger receptors or oxidative damage
**Omega-3 FA** [137,138,139]	↓ TGs probably due to: - ↓ lipogenic gene expression - ↑ β-oxidation of FA - ↑ LPL expression	1. Intrahepatic degradation of ApoB100 2.↓ ACAT2 3. Alterations in sphingolipids: - ↑ CR: VLDL1- > VLDL2- > LDL - ↓ VLDL1-ApoB100 production
**Mipomersen** [143]	Antisense oligonucleotide inhibitor of ApoB100	Selective degradation of ApoB100-mRNA
**Lomitapide** [148]	Direct inhibition of MTP in hepatocytes and enterocytes	↓ of ApoB-containing lipoproteins
**Bempedoic acid** [149,150,151]	ATP-citrate lyase inhibitor	1.↓ cholesterol synthesis in the liver 2. Up-regulation of LDL-R expression 3.↑ LDL-C clearance
**Evolocumab/** **Alirocumab** [154,155,156]	PCSK9 inhibitors	1.↓ PCSK9 ability to bind to the LDL-R: - Intrahepatic accumulation of LDL-R - ↑ LDL-C clearance - ↓ LDL-C levels 2.↓ stability and mRNA expression of ApoB
**Inclisiran** [157]	small interfering ribonucleic acid (siRNA)	↓ PCSK9-mediated degradation of LDL-R: - ↑ LDL-R expression - ↑ LDL-C uptake by the liver
**Evinacumab/** **Vupanorsen** [158,159,160]	ANGPTL3 inhibitors	- ↑ activity of LPL and EL - ↑ clearance of ApoB-containing lipoproteins
**Obicetrapib** [161,162,163]	CETP inhibitor	1.↓ the rate of transfer of CE from HDL into TLRs 2.↑ cholesterol content in HDL 3.↑ formation of larger HDL particles that are more slowly catabolized 4. Depletion of cholesterol content of ApoB

Abbreviations: ACAT2: acetyl-CoA acetyltransferase 2; ANGPTL3: angiopoietin-like 3; ApoB100: apolipoprotein B100; CETP: cholesteryl ester transfer protein; CE: cholesteryl esters; CV: conversion rate; EL: endothelial lipase; FA: fatty acids; FCR: fractional catabolic rate; HDL: high-density lipoprotein; HMG-CoA: β-Hydroxy β-methylglutaryl-CoA; LDL-C: low-density lipoprotein; LDL-R: low-density lipoprotein receptor; LPL: lipoprotein lipase; MTP: microsomal triglyceride transfer protein; NPC1L1: Niemann-Pick C1-Like 1; PPAR-α: peroxisome proliferator-activated receptor-α; PCSK9: proprotein convertase subtilisin/kexin type 9; PR: production rate; TGs: triglycerides; TLRs: triglyceride-rich lipoproteins.

## Data Availability

Not applicable.

## Abbreviations

ACAT2: acetyl-Coenzyme A acetyltransferase 2; ACCi: acetyl-CoA carboxylase inhibitor; AHA: American Heart Association; ANGPTL3: angiopoietin-like 3 protein; AMORIS: The Apoprotein-related Mortality Risk Study; APC: antigen-presenting cell;ApoA1: apolipoprotein A1; ApoB: apolipoprotein B; ApoB48: apolipoprotein B48; ApoB100: apolipoprotein B100; ApoB100-LDL: apolipoprotein B100-low-density lipoprotein; ApoB-IC: apolipoprotein B100 immune complex; ApoCII: apolipoprotein CII; ApoCIII: apolipoprotein CIII; AS: atherosclerosis; ASCVD: atherosclerotic cardiovascular disease; BA: bempedoic acid; CAD: coronary artery disease; CCS: Canadian Cardiovascular Society; CE: cholesteryl esters; CETP: cholesteryl ester transfer protein; CHD: coronary heart disease; CKD: chronic kidney disease; CVD: cardiovascular disease; CVR: conversion rate; DAMPs: danger-associated molecular patterns; DGAT1: diacylglycerol acyltransferase 1; DM: diabetes mellitus; EAS: European Atherosclerosis Society; EL: endothelial lipase; ER: endoplasmic reticulum; ESRD: end-stage renal disease; FA: fatty acid; FCR: fractional catabolic rate; FDB: familial defective apolipoprotein B100; FH: familial hypercholesterolemia; FHBL: familial hypobetalipoproteinemia; FO: fish oil; FVII: coagulation factor VII; FXI: coagulation factor XI; HA: hyperandrogenemia; HDL: high-density lipoprotein; H.E.L.P.: heparin-induced extracorporeal LDL/fibrinogen precipitation; HFD: high-fat diet; HL: hepatic lipase; HMG-CoA: β-Hydroxy β-methylglutaryl-CoA; HNF-1α: hepatic nuclear factor 1 alpha; HNF-4α: hepatic nuclear factor 4 alpha; HoFH: homozygous familial hypercholesterolemia; ICAM-1: intercellular adhesion molecule-1; IDL: intermediate-density lipoprotein; IFN-γ: interferon-γ; IL-1α: interleukin-1α; IL-1β: interleukin-1β; IL-2: interleukin-2; IL-8: interleukin-8; IL-10: interleukin-10; IL-17: interleukin-17; IL-18: interleukin-18; IL-22: interleukin-22; JAS: Japanese Atherosclerosis Society; LCAT: lecithin-cholesterol acyltransferase; LDL: low-density lipoprotein; LDL-C: low-density lipoprotein cholesterol; LDL/HDL ratio: low-density lipoprotein/high-density lipoprotein ratio; LDL-R: low-density lipoprotein receptor; LOX-1: lectin-like oxidized low-density lipoprotein receptor-1; Lp(a): lipoprotein(a); LPL: lipoprotein lipase; MAFLD: metabolic fatty liver disease; MCS-F: monocyte colony-stimulating factor; MDA: malondialdehyde; METs: metabolic syndrome; MHC: major histocompatibility complex; MI: myocardial infarction; mTOR: mammalian target of rapamycin; MTP: microsomal triglyceride transfer protein; NAFLD: non-alcoholic fatty liver disease; NASH: non-alcoholic steatohepatitis; NF-κB: nuclear factor kappa-light-chain-enhancer of activated B cells; NLPR3: NOD-like receptor protein 3; NO: nitric oxide; non-HDL-C: non-high-density lipoprotein cholesterol; NPC1L1: Niemann-Pick C1-Like 1; Ox-LDL: oxidized low-density lipoprotein; Ox-PL: oxidized phospholipids; p38: mitogen-activated protein kinase 38; PAD: peripheral artery disease; PAI-1: plasminogen activator inhibitor-1; PCOS: polycystic ovary syndrome; PCSK9: proprotein convertase subtilsin-kexin type 9; PG: proteoglycan; PLA_2_: phospholipase A_2_; PPAR-α: peroxisome proliferator-activated receptor alpha; PPAR-γ: peroxisome proliferator-activated receptor gamma; PR: production rate; PUFA: polyunsaturated fatty acid; RCT: randomized control trial; RCT: reverse cholesterol transport; ROS: reactive oxygen species; sd-LDL: small dense-LDL; siRNA: small interfering ribonucleic acid; SR-BI: scavenger receptor class B type I; STEMI: ST-segment elevation myocardial infarction; SURDIAGENE: SURvival DIAbetes and GENEtics study; T2DM: type 2 diabetes mellitus; TC: total cholesterol; TG: triglyceride; TGF-β: transforming growth factor beta; Th: T helper; Th1: T helper 1; Th17: T helper 17; TLR: Toll-like receptor; TNF-α: tumor necrosis factor-α; TPA: tissue-type plasminogen activator; TRL: triglyceride-rich lipoprotein; UPA: urokinase-type plasminogen activator; VCAM-1: vascular cell adhesion molecule; VLDL: very low-density lipoprotein.
